# Accuracy assessment of three-dimensional abutment tooth data construction using swept-source optical coherence tomography

**DOI:** 10.1371/journal.pone.0333917

**Published:** 2025-11-06

**Authors:** Mizuki Kakizawa, Hiroki Hihara, Takumi Ishikawa, Daisuke Oida, Makoto Tojo, Toru Ogawa, Kenta Shobara, Kuniyuki Izumita, Takayuki Harata, Hiroyasu Kanetaka, Nobuhiro Yoda, Masaki Hosoda, Keiichi Sasaki

**Affiliations:** 1 Division of Advanced Prosthetic Dentistry, Tohoku University Graduate School of Dentistry, Sendai, Miyagi, Japan; 2 Think-Lands Co., Ltd., Kawasaki, Knagawa, Japan; 3 Division of Comprehensive Dentistry, Tohoku University Graduate School of Dentistry, Sendai, Miyagi, Japan; 4 Dental Informatics and Radiology, Tohoku University Graduate School of Dentistry, Sendai, Miyagi, Japan; 5 Division of Dental Laboratory, Tohoku University Hospital, Sendai, Miyagi, Japan; 6 Division of Interdisciplinary Co-Creation, Liaison Center for Innovative Dentistry Tohoku University Graduate School of Dentistry, Sendai, Miyagi, Japan; 7 Department of Advanced Free Radical Science, Tohoku University Graduate School of Dentistry, Sendai, Miyagi, Japan; University of Puthisastra, CAMBODIA

## Abstract

A limitation of widely used intraoral scanners (IOSs) is their inability to capture finish lines at the subgingival marginal area, as they only extract surface information. Swept-source optical coherence tomography (SS-OCT) captures high-speed, high-resolution cross-sectional images of soft and hard tissues. Integrating this technology can overcome clinical IOS limitations. Therefore, this study was conducted to fabricate crowns from three-dimensional images scanned with SS-OCT as a proof-of-principle for its application in IOSs and to evaluate fit accuracy. TRIOS3 was used for comparison, with both SS-OCT and TRIOS3 scanned three times, and crowns were fabricated using the same digital workflow. Internal gaps were measured using scanning electron microscopy, and marginal fit was evaluated via microscopy. Results showed that TRIOS3 had superior accuracy. SS-OCT can image solely in the occlusal direction, with accuracy decreasing at greater depths, which reduces precision around the margin. Additionally, SS-OCT lacks automatic correction of surface information in computer-aided design (CAD) software. To improve SS-OCT accuracy for abutment tooth measurements, automatic margin correction, improved CAD compatibility, and specialized probes for capturing tooth features are needed.

## Introduction

Digital technology is advancing rapidly in the dental field, with intraoral scanners (IOSs) increasingly integrated into routine clinical practice. IOSs construct three-dimensional (3D) images of the dentition and occlusion without requiring impression materials or plaster models, reducing material usage and procedural time [1]. Additionally, IOSs minimize patient discomfort from impression materials and lower the risk of accidental ingestion or aspiration [2–5].

IOSs optically measure the surfaces of the target teeth and gingiva directly in the patient’s oral cavity [6], integrating the data in real-time. Accompanying software is then used to perform 3D reconstructions of the captured images [7]. However, IOSs can only capture target surface images, making it difficult to image the subgingival margin of abutment teeth. Capturing accurate impressions is also challenging when the surface is covered by saliva or blood [8]. Techniques such as gingival retraction before subgingival scanning [8,9] can mitigate these challenges but counter the advantages of IOSs, e.g., reduced procedure time and patient discomfort. Addressing these limitations could minimize the burden on patients and clinicians, broaden IOS applications, expand market demand.

To overcome these challenges, we focused on swept-source optical coherence tomography (SS-OCT), a noninvasive method that uses a micro-electromechanical systems (MEMS)–vertical-cavity surface-emitting laser as a light source and captures high-speed, high-resolution cross-sectional images of soft and hard tissues. It generates detailed images by measuring the intensity and time delay of reflected or backscattered near-infrared light using low-coherence interferometry [10]. Traditional OCT employs a super luminescent diode as a light source to obtain noninvasive tomographic images at depths of approximately 1–2 mm from tissue surfaces [11], whereas SS-OCT uses a laser to sweep oscillation frequencies linearly, calculating the reflected light intensity distribution by Fourier-transforming the interferometer’s detection signal. SS-OCT can acquire over 25 images per second with a spatial resolution of 12 μm and sweeping laser wavelengths by 100 nm [12], with OCT penetrating tissue to capture high-resolution submucosal images [13]. These capabilities suggest that SS-OCT could enable IOS to capture high-resolution subgingival margin data for abutment teeth.

SS-OCT has been researched in dentistry for caries detection [14–18], periodontal tissue observation, calculus detection [19–24], and oral cancer diagnosis [25,26]. Studies have also explored its use for 3D tooth measurements [27,28]. However, several challenges remain in applying SS-OCT to IOSs. Although 3D imaging methods using SS-OCT have been reported, no studies have evaluated dimensional accuracy. Furthermore, no research has addressed whether 3D images constructed via SS-OCT can be integrated into dental digital workflows. Therefore, a basic evaluation is essential for integrating SS-OCT into IOSs.

In this study, as a proof of concept for the application of SS-OCT to IOS, we evaluated the fabricated crown marginal and internal fit accuracy, assessing the new three-dimensional image construction method of OCT, stitching using the feature points of segmented data, which could be integrated into digital workflows by referring to prior studies [29–34]. Single abutment teeth were scanned, and crowns were fabricated using the same digital workflow with data from the SS-OCT and IOSs. The fit accuracy of the abutment teeth was then compared.

## Materials and methods

**Fig 1:**
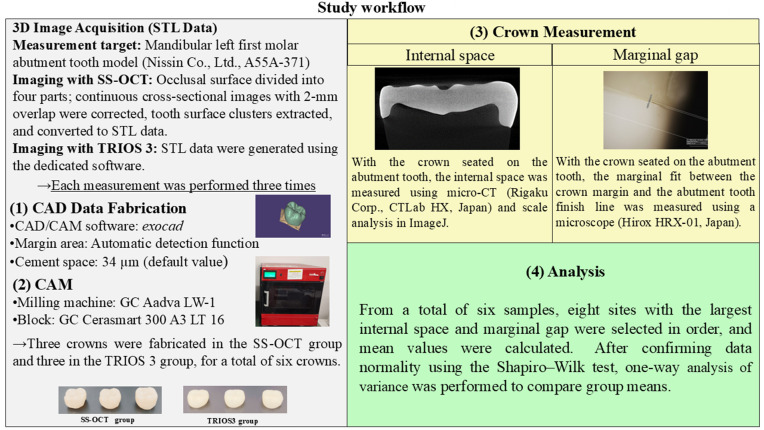
Workflow of this study.

Fig 1 shows the overall flow of the study.

### OCT device and probe

**Fig 2:**
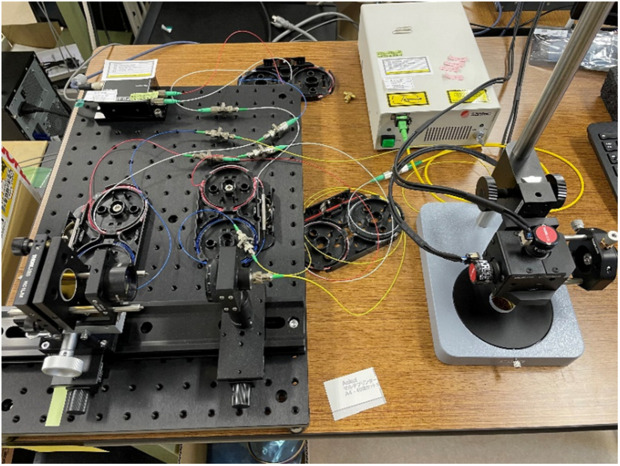
Photographs of the OCT system. The IVS-2000 served as the light source, connected to a small galvano-mirror probe.

SS-OCT with a 1310-nm wavelength and a 100-kHz scanning rate (IVS-200, Santec Holdings Corporation, Japan; Fig 2) was used as the light source. A small probe (Thorlabs Inc, USA) was used to facilitate tooth imaging. The imaging range was 7 × 7 mm, and the depth resolution was 85 μm.

### Subject

A standardized dental model for abutment teeth (A55A-371 Nissin Co., Ltd., Japan) was used in this study. Only one abutment-tooth model was used to account for the effect of size differences during fabrication.

### Control

The TRIOS3 (3Shape, Denmark) system served as the control. A clinician with over 5 years of experience performed the scanning. The resulting 3D image was converted into an STL file for comparison.

### SS-OCT scanning methods

The method involved stitching based on the feature points of the segmented data. Imaging was performed at four perpendicular locations relative to the occlusal surface. Data were stitched twice using ImageJ’s stitching function, with a field of view comprising 400 × 400 pixels (7 × 7 mm) and a 2-mm overlap (within ± 29%). The Fourier transform–based phase correlation method [35] was applied for stitching. A density-based spatial clustering of applications with noise algorithm [36] was used to set thresholds for isolating tooth surface clusters (within ±1.20 pixels), with data converted into STL files.

### 3D image acquisition

The SS-OCT method that demonstrated the best accuracy was adopted. A single operator performed three SS-OCT measurements to obtain three 3D datasets, which were converted to STL format. For comparison, a clinician with over five years of experience performed three 10-s TRIOS 3 scans (buccal, occlusal, and lingual directions) to generate three 3D datasets [6,37,38], which were also converted STL format.

### Crown fabrication

Exocad (Exocad Co., Ltd.) was used for crown data. Default settings included a 34-μm cement space and a 0-mm margin line. The software’s autodetect function identified the margin area. Computer-aided design (CAD) data were exported to a milling machine (Aadva LW-1, GC) and used with GC Cerasmart 300 A3 LT 16 (GC) CAD/computer-aided manufacturing (CAM) blocks.

### Accuracy evaluation

**Fig 3:**
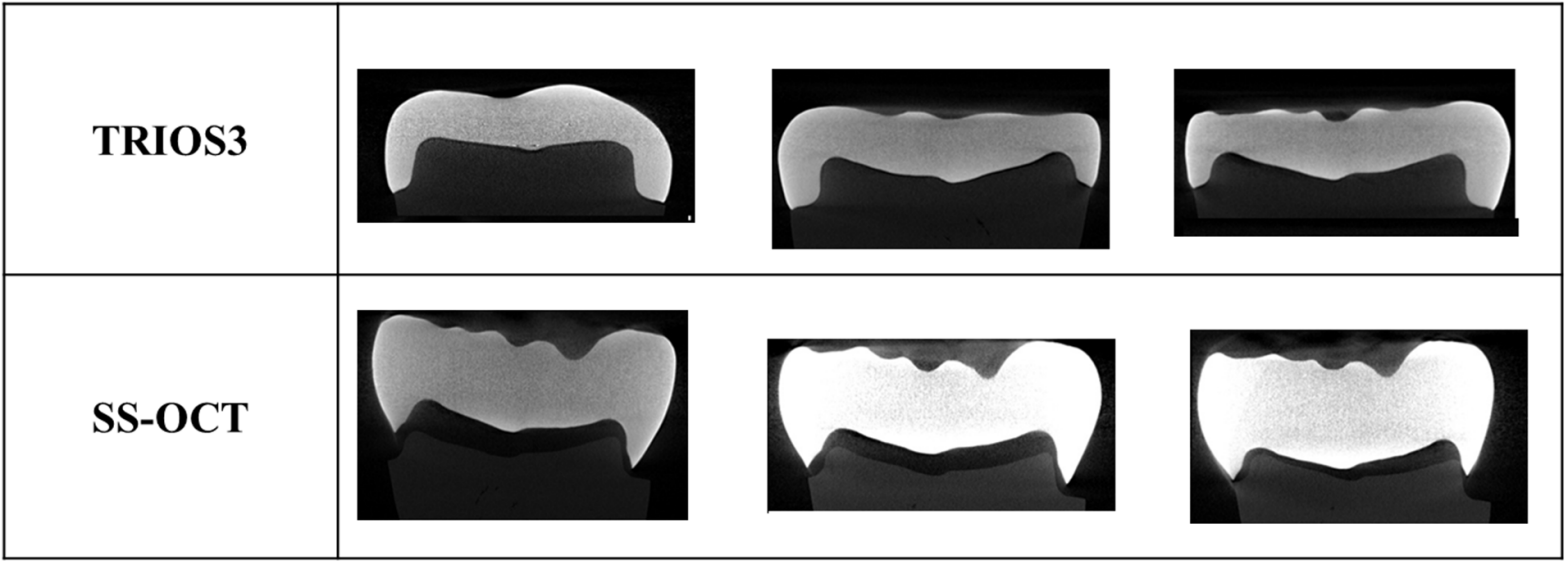
CT images showing inner spaces. Results are shown for three samples per method.

**Fig 4:**
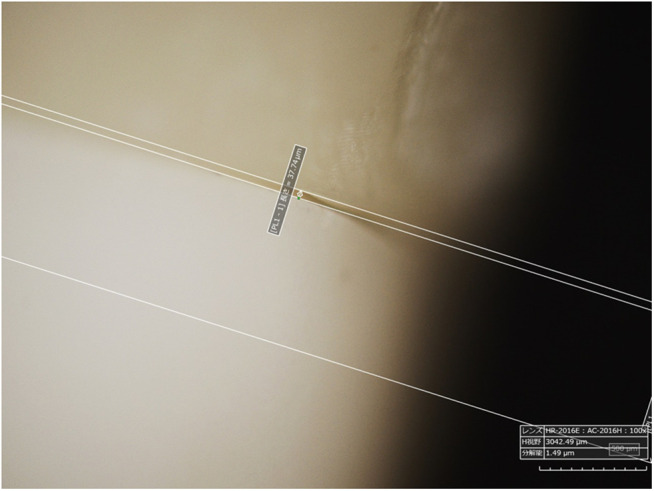
Margin gap measurement methods. Microscope Images of margin gaps. Measurements were performed using the microscope’s onboard measurement function.

Crowns were attached to abutment teeth, and computed tomography (CT) images were captured using a CT scanner (CT Lab HX, Rigaku Co., Ltd., Japan) to measure the inner space (Figs 3 and 4). Marginal fit was also evaluated using a microscope (Hirox HRX-01, Japan). For each sample, eight points with the largest inner space and margin gaps were selected. The average of these eight points was calculated to define the fit accuracy [21].

### Statistical analysis

Mean values for the eight largest inner surface spaces and margin gaps were calculated [39]. After confirming data normality using the Shapiro–Wilk test, one-way ANOVA was performed to compare group means. If the assumption of normality was met, Tukey’s post hoc test was applied for multiple comparisons. Statistical significance was set at *p* < 0.05.

## Results

**Fig 5:**
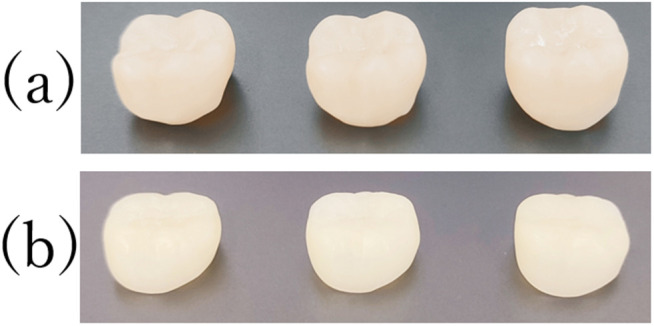
Fabricated crown. Crowns fabricated from (a) SS-OCT data and (b) TRIOS3 data.

The fabricated crowns are shown in the Fig 5. According to the Shapiro–Wilk test, the measurements of each sample followed a normal distribution. The average internal spaces for the TRIOS3 group samples were 87 ± 19, 97 ± 14, and 76 ± 21 µm, compared with 1123 ± 265, 884 ± 64, and 618 ± 255 µm for the SS-OCT group (Table 1). The mean margin gaps for these samples were 42 ± 19, 68 ± 39, and 70 ± 39 μm for the TRIOS3 group, and 1242 ± 381, 570 ± 682, and 414 ± 446 μm for the SS-OCT group, respectively (Table 2).

**Table 1:** Mean internal space gap.

	Mean internal space gap (μm)		
	Sample1	Sample2	Sample3
TRIOS3	87 ± 19	97 ± 14	76 ± 21
SS-OCT	1123 ± 265**	884 ± 64**	618 ± 255**

For occlusal inner space, samples scanned with TRIOS3 had significantly smaller spaces than Sample1, Sample2 and Sample3 scanned with SS-OCT (^**^p < 0.01). There was no significant difference within the TRIOS3. However, in SS-OCT group, Sample3 had significantly smaller space than Sample1 and Sample2 (*p < 0.01).

**Table 2:** Mean margin gap.

	Mean margin gap (μm)		
	Sample1	Sample2	Sample3
TRIOS3	42 ± 19	68 ± 39	70 ± 39
SS-OCT	1242 ± 381**	570 ± 682**	417 ± 446**

Regarding margin gaps, samples scanned with TRIOS3 had small spaces than Sample1 scanned with SS-OCT (^**^p < *0.01*). There was no significant difference within the TRIOS3 and SS-OCT group.

Regarding occlusal inner spaces, samples scanned with TRIOS3 exhibited significantly smaller spaces compared to all three SS-OCT samples (samples 1–3, *p* < 0.01). For margin gaps, TRIOS3 samples showed significantly smaller gaps compared with three SS-OCT samples (samples 1–3, *p* < 0.01). There was no significant variation within the TRIOS3 group, whereas the SS-OCT samples tended to have larger margin gaps compared with the TRIOS3 group.

## Discussion

This is the first study to fabricate crowns from SS-OCT scanning data and evaluate their accuracy. We used new 3D image construction methods and STL data conversion. Stitching using feature points relies on the Fourier transform–based phase correlation method [35]. This method estimates relative translational offsets between multiple images or datasets in the frequency domain via fast Fourier transform calculations. To mitigate global error accumulation as the number of tiles increased, enhancements were employed, including comparisons of maximum and average displacements to filter outliers and nonlinear brightness correction to adjust for tile brightness variations. These measures minimized noise and ensured greater accuracy.

Improvements in surface position extraction for STL file comparison also played a key role in enhancing accuracy. Surface noise was reduced, and clustering of point cloud data retained only clusters representing the tooth surface. This process employed the density-based spatial clustering of applications with noise algorithm [40], which identifies clusters based on two adjustable parameters: the distance to neighboring points and the minimum number of points required to define a cluster. By tuning these parameters to fit the OCT tooth images, accurate surface information was extracted and preserved.

Overall, employing feature point–based stitching combined with refined 3D image construction and surface extraction methods markedly improved imaging accuracy. This approach shows promise for producing high-quality 3D images of teeth and converting them into STL images.

Although previous studies on the assessment of crown fit to abutment teeth have evaluated the precision of intraoral 3D data by sectioning crowns and abutment teeth [32,33], the current study used a noninvasive measurement method. This approach was chosen to avoid potential distortions during section preparation and to allow for repeated measurements, ensuring reproducibility. Ferrini et al. [31] reported that the average margin error of prosthetics fabricated using TRIOS3 was 67.95 μm. Despite differences in the scanned abutment materials, our findings showed similar values, suggesting that the scanning, CAD, and CAM workflows were accurate. The margin gaps in TRIOS3-scanned samples fell within the clinically acceptable range of 120 µm defined by McLean and von Fraunhofer [41]; however, the SS-OCT–scanned samples markedly exceeded this threshold.

One likely cause of this observed discrepancy is the imaging method. IOS devices, such as TRIOS3, create 3D images by overlaying scans captured from multiple directions (e.g., occlusal, buccal, and lingual directions) [38]. Various factors, such as the probe head size, influence the imaging range, with excessive overlays potentially reducing image construction accuracy [42]. In our SS-OCT method, the 3D image was built using only occlusal images to minimize overlays. A previous study demonstrated the feasibility of constructing a 3D tooth image using only vertical SS-OCT imaging [39]. The vertical imaging range of 7 × 7 mm could theoretically capture axial surfaces, including their height. However, OCT’s lower depth resolution likely compromised imaging accuracy at the margin areas [43]. This limitation, along with vertical-only imaging, may have caused crown size differences during automatic design relative to the data obtained from TRIOS3 imaging. In our previous study, the STL files obtained from SS-OCT and TRIOS3 data were superimposed using the best-fit method and Geomagic Control X (3D Systems, USA). TRIOS3 data was used as the control. The difference from the control was calculated using the root mean square (RMS). Mean errors at four abutment corners and four margin points were also calculated [29]. The average error was 81.1 μm, which was considered to have contributed to the reduction in imaging accuracy in the margin area. To address this issue, a probe with an expanded imaging range is needed. Current SS-OCT probes differ markedly in shape from IOS probes and are unsuitable for digital impressions. Mimicking the design of existing IOS probes and downsizing them with an MEMS mirror would enhance their usability and operability in clinical practice. Additionally, SS-OCT’s sensitivity to camera shake necessitates exploring new imaging methods. Considering the success of OCT in constructing 3D images for vascular catheters [44,45], applying similar methods may improve the accuracy of SS-OCT images of teeth.

The role of the scanning software also warrants consideration. IOS devices are designed to process images based on the optical properties of the tooth structure and materials, automatically correcting and outputting margin shapes through proprietary algorithms [46]. These systems often reduce data density in noncritical areas while increasing density in marginal regions to facilitate CAD workflows [6]. In the present study, SS-OCT data was processed using general-purpose software rather than dental software. As a result, data density in the margin areas was not prioritized during STL conversion, potentially affecting accuracy. Furthermore, the performance of commercially available IOS devices has been shown to vary with software updates [46–49], suggesting that the scanning software itself could have influenced the accuracy outcomes.

Discrepancies between the software’s 3D shape accuracy verification and crown accuracy evaluations further highlight the need to consider how measurement software processes margins for STL file construction from 3D OCT images. Additionally, CAD software limitations may have contributed to inaccuracies. Automatic detection and design were used in this study, but these have previously been reported to lack precision [50]. The automated margin processing mechanism, although convenient, could represent a limitation of current CAD systems, and improved accuracy may be achievable by involving an experienced dental technician to manually define the margin line.

In recent years, IOS devices with OCT for subgingival margin detection have been developed, and their utility has been evaluated [51]. However, as image processing is performed using edge extraction (automated processing) along with Canny edge detection, followed by manual segmentation and merging using Amira and Geomagic, fully automated detection is assumed to be difficult. Meanwhile, as margin areas are still manually adjusted at the laboratory end, it is desirable that OCT-IOS systems also undergo similar manual adjustments in the laboratory.

The milling process should be examined as a contributing factor. Following crown design using the CAD software, the block placement and milling process were planned to use the milling machine’s proprietary software. Multiple factors, such as the size of available CAD/CAM blocks, disks positioning, automatic design of crown, and the milling bur’s cutting capability, can influence crown placement during fabrication. Moreover, limitations in the placement of crowns within the block [52] often complicate the milling of fine features, such as the margins and inner surfaces of the crown and size of fabricated crown, potentially affecting accuracy.

The significant differences in inner surface gap and margin gaps between TRIOSS3 and OCT may be attributed to the statistical approach and measurement variability. In addition to the small sample size, the differences were large depending on the measurement site; therefore, a normal distribution could not be assumed, and a nonparametric method was used for the test. Further validation with a larger sample size and a standardized measurement method is essential to improve accuracy and reliability.

Challenges also remain in capturing subgingival margins, the primary goal of the developed device. First, full-arch scanning accuracy must be validated, as current validation is limited to a single tooth. Existing IOS data show that full-arch scanning accuracy is lower than that of traditional impression materials [53–55]. Therefore, further research is necessary on stitching methods for full-arch imaging.

As OCT is partially absorbed by biological tissues, it is also crucial to consider the effects of blood and bodily fluids. The refractive index of human gingiva must also be evaluated, as SS-OCT is affected by gingival refraction. The refractive index of human oral gingiva is 1.41 [51], and *in vitro* evaluation is possible by using silicone, which exhibits a similar refractive index. However, values for molars and inflamed gingiva remain unknown. Animal or clinical studies are needed to collect these data. Addressing these issues could lead to valuable clinical applications.

In clinical practice, one of the constraints to consider is that SS-OCT is expensive. To overcome this limitation, fundamental developments, such as the creation of low-cost light sources, are necessary. Addressing this issue could lead to the commercialization of this technology.

## Conclusions

In conclusion, it was possible to fabricate crowns from 3D images acquired with SS-OCT. However, for clinical application, further improvements are needed in the stitching method for constructing 3D images to increase dimensionality. Additionally, from a software perspective, it is necessary to sharpen the information in critical areas of dental prosthesis design and to improve the compatibility of these areas with CAD/CAM workflows. From a hardware perspective, downsized probes that can be adapted to the oral cavity are needed. These advancements would enable the creation of a new generation of IOS systems capable of capturing highly accurate measurements of the full arch of the dentition, including those of the gingival margin, and in a challenging oral environment, including the presence of blood and saliva.

## References

[1] Mangano F, Gandolfi A, Luongo G, Logozzo S. Intraoral scanners in dentistry: a review of the current literature. BMC Oral Health. 2017;17(1):149. doi: 10.1186/s12903-017-0442-x 29233132 PMC5727697

[2] Joda T, Brägger U. Patient-centered outcomes comparing digital and conventional implant impression procedures: a randomized crossover trial. Clin Oral Implants Res. 2016;27(12):e185–9. doi: 10.1111/clr.12600 25864771

[3] Haddadi Y, Bahrami G, Isidor F. Evaluation of Operating Time and Patient Perception Using Conventional Impression Taking and Intraoral Scanning for Crown Manufacture: A Split-mouth, Randomized Clinical Study. Int J Prosthodont. 2018;31(1):55–9. doi: 10.11607/ijp.5405 29145527

[4] Yuzbasioglu E, Kurt H, Turunc R, Bilir H. Comparison of digital and conventional impression techniques: evaluation of patients’ perception, treatment comfort, effectiveness and clinical outcomes. BMC Oral Health. 2014;14:10. doi: 10.1186/1472-6831-14-10 24479892 PMC3913616

[5] Ahlholm P, Sipilä K, Vallittu P, Jakonen M, Kotiranta U. Digital Versus Conventional Impressions in Fixed Prosthodontics: A Review. J Prosthodont. 2018;27(1):35–41. doi: 10.1111/jopr.12527 27483210

[6] Hotta Y. Basic knowledge of 3D optical measurement methods used for intraoral scanners. Ann Jpn Prosthodont Soc. 2021;13:291–8.

[7] Richert R, Goujat A, Venet L, Viguie G, Viennot S, Robinson P, et al. Intraoral Scanner Technologies: A Review to Make a Successful Impression. J Healthc Eng. 2017;2017:8427595. doi: 10.1155/2017/8427595 29065652 PMC5605789

[8] Casucci A, Verniani G, Habib R, Ricci NM, Carboncini C, Ferrari M. Accuracy of Four Intra-Oral Scanners in Subgingival Vertical Preparation: An In Vitro 3-Dimensional Comparative Analysis. Materials (Basel). 2023;16(19):6553. doi: 10.3390/ma16196553 37834690 PMC10574066

[9] Sanda K, Yasunami N, Matsushita Y, Furuhashi A, Matsuzaki T, Imai M, et al. Trueness of the Combined Intra- and Extraoral Scanning Technique for Transferring Subgingival Contours from Provisional Restorations to Definitive Restorations. Int J Prosthodont. 2023;36(3):308–14. doi: 10.11607/ijp.7030 36484679

[10] Putra RH, Yoda N, Astuti ER, Sasaki K. Potential imaging capability of optical coherence tomography as dental optical probe: a mini-review. Appl Sci. 2021;11:11025.

[11] Aumann S, Donner S, Fischer J, Müller F. Chapter 3: optical coherence tomography (OCT): principle and technical realization. In: High resolution imaging in microscopy and ophthalmology: new frontiers in biomedical optics. Berlin: Springer; 2019: 59–85. 32091846

[12] Laíns I, Wang JC, Cui Y, Katz R, Vingopoulos F, Staurenghi G, et al. Retinal applications of swept source optical coherence tomography (OCT) and optical coherence tomography angiography (OCTA). Prog Retin Eye Res. 2021;84:100951. doi: 10.1016/j.preteyeres.2021.100951 33516833

[13] Machoy M, Seeliger J, Szyszka-Sommerfeld L, Koprowski R, Gedrange T, Woźniak K. The Use of Optical Coherence Tomography in Dental Diagnostics: A State-of-the-Art Review. J Healthc Eng. 2017;2017:7560645. doi: 10.1155/2017/7560645 29065642 PMC5534297

[14] Shimada Y, Sadr A, Burrow MF, Tagami J, Ozawa N, Sumi Y. Validation of swept-source optical coherence tomography (SS-OCT) for the diagnosis of occlusal caries. J Dent. 2010;38(8):655–65. doi: 10.1016/j.jdent.2010.05.004 20470855

[15] Nakagawa H, Sadr A, Shimada Y, Tagami J, Sumi Y. Validation of swept source optical coherence tomography (SS-OCT) for the diagnosis of smooth surface caries in vitro. J Dent. 2013;41(1):80–9. doi: 10.1016/j.jdent.2012.10.007 23084870

[16] Zhou Y, Shimada Y, Matin K, Sadr A, Yoshiyama M, Sumi Y, et al. Assessment of root caries under wet and dry conditions using swept-source optical coherence tomography (SS-OCT). Dent Mater J. 2018;37(6):880–8. doi: 10.4012/dmj.2017-273 29962412

[17] Ei TZ, Shimada Y, Abdou A, Sadr A, Yoshiyama M, Sumi Y, et al. Three-dimensional assessment of proximal contact enamel using optical coherence tomography. Dent Mater. 2019;35(4):e74–82. doi: 10.1016/j.dental.2019.01.008 30770133

[18] Kitasako Y, Sadr A, Shimada Y, Ikeda M, Sumi Y, Tagami J. Remineralization capacity of carious and non-carious white spot lesions: clinical evaluation using ICDAS and SS-OCT. Clin Oral Investig. 2019;23(2):863–72. doi: 10.1007/s00784-018-2503-1 29948272

[19] Lee C, Darling CL, Fried D. Polarization-sensitive optical coherence tomographic imaging of artificial demineralization on exposed surfaces of tooth roots. Dent Mater. 2009;25(6):721–8. doi: 10.1016/j.dental.2008.11.014 19167052 PMC2701248

[20] Hsieh Y-S, Ho Y-C, Lee S-Y, Lu C-W, Jiang C-P, Chuang C-C, et al. Subgingival calculus imaging based on swept-source optical coherence tomography. J Biomed Opt. 2011;16(7):071409. doi: 10.1117/1.3602851 21806255

[21] Kao M-C, Lin C-L, Kung C-Y, Huang Y-F, Kuo W-C. Miniature endoscopic optical coherence tomography for calculus detection. Appl Opt. 2015;54(24):7419–23. doi: 10.1364/AO.54.007419 26368780

[22] Park J-Y, Chung J-H, Lee J-S, Kim H-J, Choi S-H, Jung U-W. Comparisons of the diagnostic accuracies of optical coherence tomography, micro-computed tomography, and histology in periodontal disease: an ex vivo study. J Periodontal Implant Sci. 2017;47(1):30–40. doi: 10.5051/jpis.2017.47.1.30 28261522 PMC5332333

[23] Tsubokawa M, Aoki A, Kakizaki S, Taniguchi Y, Ejiri K, Mizutani K, et al. In vitro and clinical evaluation of optical coherence tomography for the detection of subgingival calculus and root cementum. J Oral Sci. 2018;60(3):418–27. doi: 10.2334/josnusd.17-0289 29794398

[24] Kakizaki S, Aoki A, Tsubokawa M, Lin T, Mizutani K, Koshy G, et al. Observation and determination of periodontal tissue profile using optical coherence tomography. J Periodontal Res. 2018;53(2):188–99. doi: 10.1111/jre.12506 29063599

[25] Wilder-Smith P, Krasieva T, Jung W-G, Zhang J, Chen Z, Osann K, et al. Noninvasive imaging of oral premalignancy and malignancy. J Biomed Opt. 2005;10(5):051601. doi: 10.1117/1.2098930 16292949

[26] Tsai M-T, Lee H-C, Lu C-W, Wang Y-M, Lee C-K, Yang CC, et al. Delineation of an oral cancer lesion with swept-source optical coherence tomography. J Biomed Opt. 2008;13(4):044012. doi: 10.1117/1.2960632 19021340

[27] Eom JB, Ahn JS, Eom J, Park A. Wide field of view optical coherence tomography for structural and functional diagnoses in dentistry. J Biomed Opt. 2018;23(7):1–8. doi: 10.1117/1.JBO.23.7.076008 30008193

[28] Kashiwa M, Shimada Y, Sadr A, Yoshiyama M, Sumi Y, Tagami J. Diagnosis of Occlusal Tooth Wear Using 3D Imaging of Optical Coherence Tomography Ex Vivo. Sensors (Basel). 2020;20(21):6016. doi: 10.3390/s20216016 33113981 PMC7660331

[29] Chiu A, Chen Y-W, Hayashi J, Sadr A. Accuracy of CAD/CAM Digital Impressions with Different Intraoral Scanner Parameters. Sensors (Basel). 2020;20(4):1157. doi: 10.3390/s20041157 32093174 PMC7071446

[30] Cho S-H, Schaefer O, Thompson GA, Guentsch A. Comparison of accuracy and reproducibility of casts made by digital and conventional methods. J Prosthet Dent. 2015;113(4):310–5. doi: 10.1016/j.prosdent.2014.09.027 25682531

[31] Ferrini F, Sannino G, Chiola C, Capparé P, Gastaldi G, Gherlone EF. Influence of Intra-Oral Scanner (I.O.S.) on The Marginal Accuracy of CAD/CAM Single Crowns. Int J Environ Res Public Health. 2019;16(4):544. doi: 10.3390/ijerph16040544 30769768 PMC6406818

[32] Sannino G, Gloria F, Schiavetti R, Ottria L, Barlattani A. Dental Wings CAD/CAM system precision: an internal and marginal fit sperimental analisys. Oral Implantol (Rome). 2009;2(3):11–20. 23285364 PMC3415347

[33] Dauti R, Cvikl B, Franz A, Schwarze UY, Lilaj B, Rybaczek T, et al. Comparison of marginal fit of cemented zirconia copings manufactured after digital impression with lava™ C.O.S and conventional impression technique. BMC Oral Health. 2016;16(1):129. doi: 10.1186/s12903-016-0323-8 27931256 PMC5146899

[34] Rödiger M, Schneider L, Rinke S. Influence of Material Selection on the Marginal Accuracy of CAD/CAM-Fabricated Metal- and All-Ceramic Single Crown Copings. Biomed Res Int. 2018;2018:2143906. doi: 10.1155/2018/2143906 29765979 PMC5885340

[35] Preibisch S, Saalfeld S, Tomancak P. Globally optimal stitching of tiled 3D microscopic image acquisitions. Bioinformatics. 2009;25(11):1463–5. doi: 10.1093/bioinformatics/btp184 19346324 PMC2682522

[36] Ester M, Kriegel HP, Sander J, Xu X. A density-based algorithm for discovering clusters in large spatial databases with noise. In: Proceedings of the Second International Conference on Knowledge Discovery and Data Mining (KDD’96), 1996. 226–31.

[37] Revilla-León M, Sicilia E, Agustín-Panadero R, Gómez-Polo M, Kois JC. Clinical evaluation of the effects of cutting off, overlapping, and rescanning procedures on intraoral scanning accuracy. J Prosthet Dent. 2023;130(5):746–54. doi: 10.1016/j.prosdent.2021.10.017 34998582

[38] Zimmermann M, Valcanaia A, Neiva G, Mehl A, Fasbinder D. Three-Dimensional Digital Evaluation of the Fit of Endocrowns Fabricated from Different CAD/CAM Materials. J Prosthodont. 2019;28(2):e504–9. doi: 10.1111/jopr.12770 29508488

[39] Nakajima Y, Shimada Y, Sadr A, Wada I, Miyashin M, Takagi Y, et al. Detection of occlusal caries in primary teeth using swept source optical coherence tomography. J Biomed Opt. 2014;19(1):16020. doi: 10.1117/1.JBO.19.1.016020 24474506

[40] Moon S, Chen Z. Phase-stability optimization of swept-source optical coherence tomography. Biomed Opt Express. 2018;9(11):5280–95. doi: 10.1364/BOE.9.005280 30460128 PMC6238911

[41] McLean JW, von Fraunhofer JA. The estimation of cement film thickness by an in vivo technique. Br Dent J. 1971;131:107–11. 5283545 10.1038/sj.bdj.4802708

[42] Abe M, Kondo H, Tanabe N, Sato H, Fukutoku AS. Effects of intraoral scanner head size on the accuracy of image data reproduction. Ann Jpn Prosthodont Soc. 2023;15:211–8.

[43] Ng J, Ruse D, Wyatt C. A comparison of the marginal fit of crowns fabricated with digital and conventional methods. J Prosthet Dent. 2014;112(3):555–60. doi: 10.1016/j.prosdent.2013.12.002 24630399

[44] Huo L, Xi J, Wu Y, Li X. Forward-viewing resonant fiber-optic scanning endoscope of appropriate scanning speed for 3D OCT imaging. Opt Express. 2010;18(14):14375–84. doi: 10.1364/OE.18.014375 20639922 PMC3408911

[45] Zhang J, Nguyen T, Potsaid B, Jayaraman V, Burgner C, Chen S, et al. Multi-MHz MEMS-VCSEL swept-source optical coherence tomography for endoscopic structural and angiographic imaging with miniaturized brushless motor probes. Biomed Opt Express. 2021;12(4):2384–403. doi: 10.1364/BOE.420394 33996236 PMC8086463

[46] Schmalzl J, Róth I, Borbély J, Hermann P, Vecsei B. The impact of software updates on accuracy of intraoral scanners. BMC Oral Health. 2023;23(1):219. doi: 10.1186/s12903-023-02926-y 37061664 PMC10105929

[47] Schmidt A, Klussmann L, Wöstmann B, Schlenz MA. Accuracy of Digital and Conventional Full-Arch Impressions in Patients: An Update. J Clin Med. 2020;9(3):688. doi: 10.3390/jcm9030688 32143433 PMC7141355

[48] Gracis S, Appiani A, Noè G. Digital workflow in implant prosthodontics: The critical aspects for reliable accuracy. J Esthet Restor Dent. 2023;35(1):250–61. doi: 10.1111/jerd.13004 36606714

[49] Zarauz C, Pradíes GJ, Chebib N, Dönmez MB, Karasan D, Sailer I. Influence of age, training, intraoral scanner, and software version on the scan accuracy of inexperienced operators. J Prosthodont. 2023;32(S2):135–41. doi: 10.1111/jopr.13785 37837217

[50] Ye J, Wang S, Wang Z, Liu Y, Sun Y, Ye H, et al. Comparison of the dimensional and morphological accuracy of three-dimensional digital dental casts digitized using different methods. Odontology. 2023;111(1):165–71. doi: 10.1007/s10266-022-00736-2 36068382

[51] Son K, Lee W, Kim W-T, Jeon M, Kim J, Jin M-U, et al. A feasibility study on the use of an intraoral optical coherence tomography system for scanning the subgingival finish line for the fabrication of zirconia crowns: An evaluation of the marginal and internal fit. J Dent. 2024;151:105386. doi: 10.1016/j.jdent.2024.105386 39366541

[52] Mota EG, Smidt LN, Fracasso LM, Burnett LHJ, Spohr AM. The effect of milling and postmilling procedures on the surface roughness of CAD/CAM materials. J Esthet Restor Dent. 2017;29:450–8. 28891600 10.1111/jerd.12326

[53] Cordaro M, Sailer I, Zarauz C, Liu X, Karasan D. The Accuracy of Full-Arch Intraoral Optical Impressions (IOS): Clinical Pilot Study of the Influence of the Scan Strategy, Operator, and Intraoral Scanner. Int J Prosthodont. 2023;36(6):689–96. doi: 10.11607/ijp.8113 38109389

[54] Ke Y, Zhang Y, Wang Y, Chen H, Sun Y. Comparing the accuracy of full-arch implant impressions using the conventional technique and digital scans with and without prefabricated landmarks in the mandible: An in vitro study. J Dent. 2023;135:104561. doi: 10.1016/j.jdent.2023.104561 37236297

[55] Cheng J, Zhang H, Liu H, Li J, Wang HL, Tao X. Accuracy of edentulous full-arch implant impression: an in vitro comparison between conventional impression, intraoral scan with and without splinting, and photogrammetry. Clin Oral Implants Res. 2024;35: 560–72. 38421115 10.1111/clr.14252

